# Water Quality and Herbivory Interactively Drive Coral-Reef Recovery Patterns in American Samoa

**DOI:** 10.1371/journal.pone.0013913

**Published:** 2010-11-10

**Authors:** Peter Houk, Craig Musburger, Phil Wiles

**Affiliations:** 1 Pacific Marine Resources Institute, Saipan, Commonwealth of the Northern Mariana Islands; 2 Department of Zoology, University of Hawai'i at Manoa, Honolulu, Hawaii, United States of America; 3 American Samoa Environmental Protection Agency, Pago Pago, American Samoa; California Academy of Sciences, United States of America

## Abstract

**Background:**

Compared with a wealth of information regarding coral-reef recovery patterns following major disturbances, less insight exists to explain the cause(s) of spatial variation in the recovery process.

**Methodology/Principal Findings:**

This study quantifies the influence of herbivory and water quality upon coral reef assemblages through space and time in Tutuila, American Samoa, a Pacific high island. Widespread declines in dominant corals (*Acropora* and *Montipora*) resulted from cyclone Heta at the end of 2003, shortly after the study began. Four sites that initially had similar coral reef assemblages but differential temporal dynamics four years following the disturbance event were classified by standardized measures of ‘recovery status’, defined by rates of change in ecological measures that are known to be sensitive to localized stressors. Status was best predicted, interactively, by water quality and herbivory. Expanding upon temporal trends, this study examined if similar dependencies existed through space; building multiple regression models to identify linkages between similar status measures and local stressors for 17 localities around Tutuila. The results highlighted consistent, interactive interdependencies for coral reef assemblages residing upon two unique geological reef types. Finally, the predictive regression models produced at the island scale were graphically interpreted with respect to hypothesized site-specific recovery thresholds.

**Conclusions/Significance:**

Cumulatively, our study purports that moving away from describing relatively well-known patterns behind recovery, and focusing upon understanding causes, improves our foundation to predict future ecological dynamics, and thus improves coral reef management.

## Introduction

Poor water quality and reduced herbivory often represent the greatest impediments to favorable coral-reef recovery following large-scale disturbance events [1]–[4]. Indeed, studies that forecast increasing disturbance frequencies due to climate induced change often end in recommendations to address local stressors to facilitate resiliency through time [5], [6]. However, limited insight exists to identify when thresholds may be crossed, and to quantify if stressors exceed the conditions necessary for (optimal) disturbance-recovery cycles.

A wealth of manipulative studies using individual organisms (i.e., corals) or small plots of reef have documented negative impacts to reef assemblages where herbivory and water quality are reduced [7]–[10]. Decreased coral and fish species richness, increasing dominance of assemblages by fewer species, increased macroalgal abundance, and even permanent phase shifts from coral to algal have been reported. However, the imposed experimental conditions constitute extreme environments that may not be prevalent (i.e., complete herbivore exclusion or continuous fertilization), especially throughout the Pacific [11]. Therefore, a disconnect between observable change reported by manipulative experiments and ecological change observed across reefscapes emerges. Beyond manipulative conditions, this disconnect may also be a result of the spatial scale of investigation [12]–[15]. Levin [16] showed that when increasing the complexity of study designs, defined by the number, strength, and scale of ecological interactions, contrasting patterns often become evident.

In order to improve our understanding and prediction of environmental thresholds leading to undesirable change on coral reefs, it seems logical to draw upon evidence collected at spatial scales appropriate to observe reef assemblages through time (∼100–500 m^2^), matching the majority of existing management, policy, and perception. Yet, this is a difficult task requiring multi-year investigations that encompass both ecological and environmental datasets. Understandably then few studies have defined relationships between environmental thresholds and observable ecological change on coral reefs because sampling rarely occurs along a gradient of environmental conditions through time, a required basis [17].

In lieu of multi-year datasets, many studies investigating coral reef assemblages at ecological scales have compared sites from contrasting environmental regimes (i.e., heavily populated versus unpopulated regions or high versus low nutrient concentrations), and corroborate the nature and magnitude of the cause(s) that underpin ‘undesirable’ ecological states [18]–[20]. In these situations differences in reef assemblages are due to longer-term, integrated responses of ecological assemblages to environmental conditions, and therefore, statistical confirmations of the cause(s) that led to ‘undesirable’ conditions are not available [19]–[21]. Despite much logical insight gained, the differences in natural history and disturbance regimes between locales remain critical, and are difficult to account for.

In instances where studies have extended through disturbance and recovery periods contrasting ecological recovery patterns, their causes, and their mechanisms have been reported [22]–[25]. However, examples remain limited, especially in the Pacific where global coral diversity peaks [26], and a strong social, cultural, and economic reliance on coral reefs perpetuates. Supporting statistics from the Science Citation Index search engine reveal published studies have a heavy focus upon herbivory, and no-take protected areas, when investigating recovery patterns on coral reefs. Few studies interactively consider localized stressors and conduct investigation along naturally occurring environmental gradients. Thus, our collective insight surrounding environmental thresholds and ecological dynamics on coral reefs remains limited.

**Figure 1:**
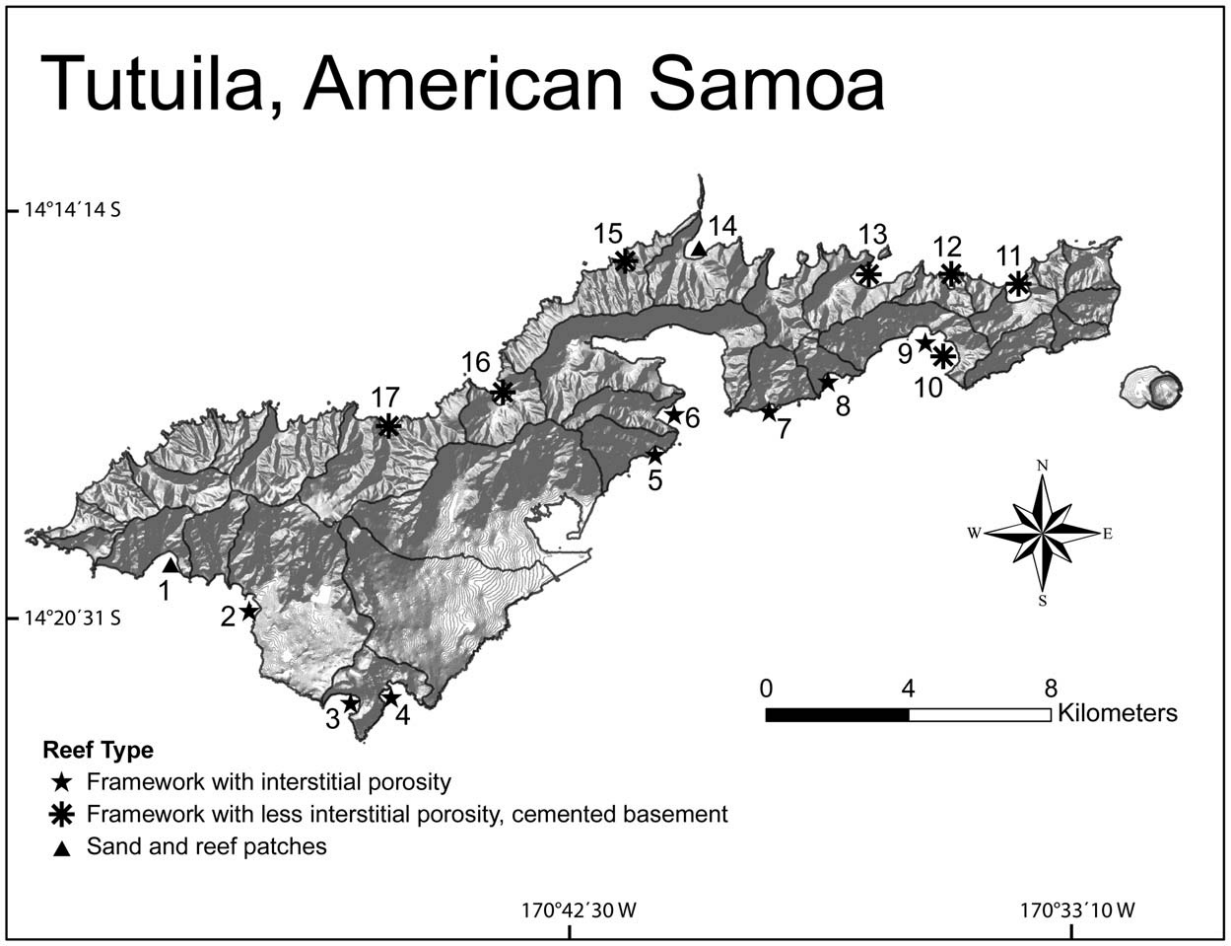
A map of the study area with site-symbols indicating geomorphological reef type, defined in Materials and Methods.

Here, we build upon the doctrine and examine the causes of differential ecological recovery using multi-year datasets from seventeen locations around Tutuila, American Samoa (Figure 1). We first determine the general impacts of a large-scale disturbance associated with tropical cyclone Heta, and isolate upon four sites where similar coral reef assemblages existed, but differential dynamics through time were noted. Subsequently, herbivory, water quality, and their interaction were tested for their ability to predict ‘favorable’ dynamics, defined within by establish metrics of coral reef assemblages sensitive to localized stressors. Expanding upon temporal trends, predictive regressions between coral reef assemblages and local stressors were defined spatially, across a gradient of 17 sites around Tutuila. Lastly, we integrate the spatial and temporal findings to form a logical basis for management and future direction.

## Results

**Figure 2:**
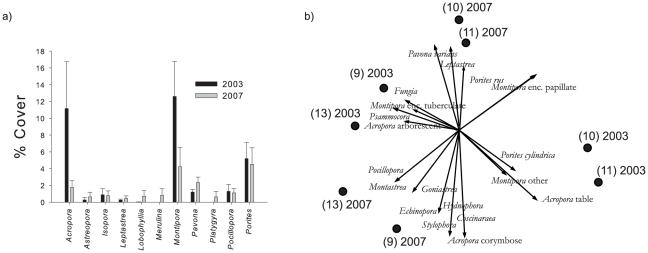
Change in coral assemblages before and after Cyclone Heta. Benthic transect data highlight a consistent decline in *Montipora* and *Acropora* corals for all monitoring sites combined (±SE) (a). Four sites that initially held similar assemblages were analyzed through time using principle component analysis of quadrat-based, colony-size data (b). Site numbers refer to Fig. 1. The horizontal axis accounts for 48.5% of the variation in species abundances, the vertical axis accounts for 18.3%.

Tropical cyclone Heta was the most plausible explanation for the observed widespread decline in coral abundance that was evident between 2003 and 2005. Coral loss following this disturbance ranged between 5 to 45%, and was heavily dependent upon the initial coral community composition, prior to the cyclone. Nearly 90% of the decline in coral cover was attributed to the loss of table and corymbose *Acropora*, and encrusting and plate *Montipora* (Figure 2a). Most notable to the present study, four sites that had initial assemblages with high *Acropora* and *Montipora* abundances showed differential recovery four years after the disturbance. Two sites had non-significant changes in favorable benthic substrate and coral species richness (sites 9 and 13, Figure 1; i.e., ‘high recovery’, defined in methods), while two had significant declines (10 and 11, Figure 1; i.e., ‘low recovery’). Turf and inhibitive coralline algae became dominant at ‘low recovery’ sites (Figure S1), and coral demography was most dynamic. Decreased colony size distributions became evident after the disturbance where very poor water quality but moderate herbivory existed (site 10, Figure S2, P<0.005, Kolmogorov-Smirnov cumulative frequency tests). In contrast, a growth of surviving *Porites rus* and *Pavona varians* corals to larger sizes were found where moderate water quality but very low herbivory existed (site 11). Multivariate assemblage data highlighted similar directional shifts in dominance to *Pavona varians* and other *Porites* colonies (mainly *P. lichen*, and *P. rus*, Figure 2b, species-centered PCA, horizontal axis explained 48.5% of the dataset variance, vertical axis explained 18.3%). Finally, coral community evenness (estimated by Margalef's d-statistic) remained significantly lower 3.5 years after the disturbance (paired t-test, coral abundances from replicate benthic transect data, P<0.05).

In contrast, non-significant trends in benthic substrate ratio's were evident at ‘high recovery’ sites (9 and 13). Coral colony size distributions were less dynamic, species richness did not significantly change, and evenness increased between 2003 and 2007 (paired t-test, P<0.01). These sites initially had high abundances of *Acropora* and *Montipora* corals, however coral assemblages shifted their dominance to *Pocillopora*, *Stylophora*, numerous faviid corals, as well juvenile corymbose *Acropora* by 2007.

**Figure 3:**
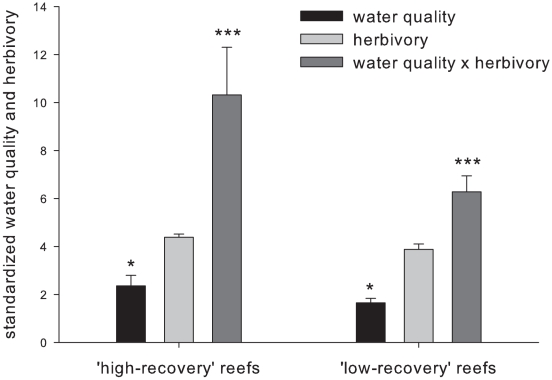
Results from pairwise testing of water quality and herbivory between ‘high’ and ‘low’ recovery reefs, defined by sensitive ecological metrics (*see Materials and Methods*). Several water quality parameters were ranked and combined to form the index reported here; higher values refer to better water quality (*see* methods). P-values are as follows: (*P<0.05, **P<0.01, and ***P<0.001).

Water quality and herbivorous fish biomass were significant predictors of recovery. Higher water quality indices were noted on ‘high recovery’ reefs compared with ‘low’ (P<0.05, paired t-test, Figure 3). While herbivory alone was not significantly different between categories, the interaction term (*water quality × herbivory*) was the best predictor (P<0.001, paired t-test, Figure 3). Notably, extremely low abundances of grazing urchins were consistently found on all reefs (<0.005 per m^2^, long term average), and did not explain any proportion of the variance in reef status.

Multiple regression analyses found strong consistencies between island gradients and temporal, site-specific trends noted above. Models that significantly explained the variance in coral cover consistently incorporated long-term environmental constraints, watershed size and wave exposure (R^2^ values between 0.66 and 0.78, Table 1). In contrast, both the benthic substrate ratio and coral species richness were best predicted by disturbed land, human population, and herbivorous fish (R^2^ values between 0.59 and 0.99, Table 1). For coral assemblages residing upon primary framework reefs with interstitial spaces common throughout the reef matrix, found mainly on the south side of Tutuila (reef type 1), wave exposure was clearly a primary explanatory variable. However, best-fit models always included local stressors. These findings are consistent with wave model summaries showing greatest exposure associated with the prevailing southeast trade winds (Table S1). Clearly measures such as coral species richness and benthic substrate ratio were sensitive to proxies of pollution and herbivory while coral cover was most dependent upon natural environmental regimes.

**Table 1:** Results from regression models describing how well driving independent variables predicted relevant ecological metrics for two unique reef types, described in Materials and Methods.

Framework reefs with lower interstitial porosity, cemented basement										
*Dependent*	*Model Fit*	*Variables*	*Slope (1)*	*SE*	*Slope (2)*	*SE*	*Intercept*	*R^2^*	*P-Value*	*AIC*
Benthic substrate ratio	1	dist land^−0.6^	7.94	1.73	—	—	−2.36	0.77	0.005	5.4
Benthic substrate ratio	2	human pop	−0.47	0.15	—	—	3.12	0.59	0.02	9.3
Coral species richness	1	herb fish + dist land	2.49	0.42	−1.89	0.46	22.89	0.92	0.003	23.5
Coral species richness	2	herb fish + human pop	2.39	0.44	−1.71	0.44	23.32	0.91	0.003	24.2
Coral cover	1	shed size^10^ × exposure^−3.2^	−2.8e-05	7.9e-06	—	—	37.9	0.66	0.01	52.4

Framework reefs with higher interstitial porosity										
Benthic substrate ratio	1	Exposure^25^ + herb fish	−7.6e-18	3.2e-19	.28	.03	1.94	0.99	<0.001	−10.3
Benthic substrate ratio	2	(exposure^25^ × human pop^0.51^) + herb fish	−3.3e-18	4.9e-19	.33	.11	1.69	0.89	0.01	4.4
Coral species richness	1	Exposure^25^ + herb fish	−7.3e-17	1.3e-17	4.7	1.3	21.7	0.87	0.02	34.2
Coral species richness	2	(exposure^25^ × dist land^−1.2^) + herb fish	−3.2e-16	7.5e-17	4.2	1.6	21.7	0.79	0.04	37.2
Coral cover	2	exposure^25^ × shed size^−0.09^	−1.2e-16	2.7e-17	—	—	41.2	0.78	0.01	41.7

**Figure 4:**
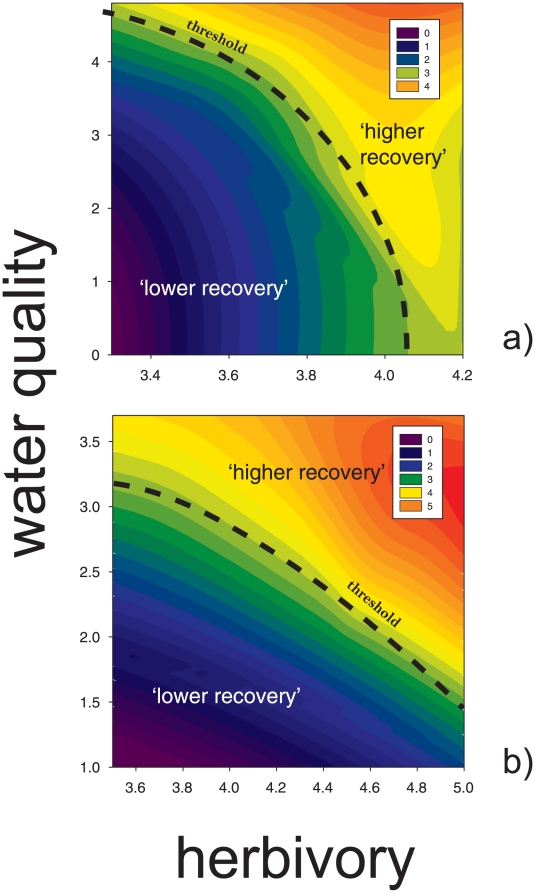
Contour plots generated from predictive multiple regression models that defined relationships between localized stressors and reef ‘status’. The black dashed lines indicate herbivory and water quality thresholds for ‘desirable’ disturbance-recovery cycles based upon the temporal examinations of four sites. Larger axis values indicate better water quality and more herbivorous fish (*see* methods). Plots are shown for framework reefs with interstitial spaces common throughout the reef matrix, found mainly on the south side of Tutuila (a), and primary framework reefs with a well-cemented, underlying basement lacking significant interstitial space development, found mainly on the north side of the island (b).

Regression models provided a basis for the interpolation of recovery status with respect to local stressors at the island scale, while categorical response trends for four sites defined a hypothesized threshold for the persistence of favorable recovery cycles through time (Figure 4a–b). Interpolation plots also identified synergistic dependencies between water quality, herbivory, and recovery status. These findings were evident for assemblages residing upon both reef types where primary coral framework is dominant; however the relative influence of water quality and herbivory became more apparent. For framework reefs found mainly on the south side of Tutuila, one unit change in water quality was more influential compared to one unit change in herbivory (Figure 4a). In contrast, herbivory was more influential for reefs typically found on the north side of the island (Figure 4b).

## Discussion

Intriguing evidence presented here shows that water quality and herbivory interactively accounted for the temporal and spatial variances associated with ‘favorable’ coral assemblage dynamics in American Samoa. Clearly further efforts will serve to refine our predictions as they pertain to Pacific coral reefs, however, the results are among the first to collectively quantify thresholds in localized stressors that are associated with ecological status. It is notable that ‘desirable’ ecological status, as defined here, was not always represented by the recovery of coral assemblages to their pre-existing states. Rather the results agree that intermediate ecological assemblages may arise following disturbance [27]–[30]. While shifting assemblages are often perceived as indicators of reduced recovery; they also represent probable mechanisms to maintain ecosystem function following disturbance [30]. In reconciliation of the two viewpoints, we reported shifting assemblages under both ‘favorable’ and ‘unfavorable’ recovery scenarios, with favorability defined by standardized ecological metrics that had affinities with water quality and herbivory through space and time. In support, previous studies have reported ‘favorable’ post-bleaching recovery in a temporal and taxonomic manner consistent with the present findings [24]. We conclude that standardized rates of change in coral species richness and benthic substrates were ideal indicators of ecological status on coral reefs, but further, attributing status to measurable environmental regimes represents a desirable means towards identifying thresholds relevant for management.

When investigations were expanded to the island scale contrasting hierarchical influences of local stressors were reported in accordance with geological reef structure. The suspect driver of these trends is the varying degree of freshwater seepage through the volcanic island. Ambient salinity levels are typically higher on the southern side of Tutuilia [31], and the noted differences have previously been linked with ecologically distinct reef assemblages [32], [33]. Enhanced water quality profiling would not only improve our understanding of natural constraints limiting coral assemblages [34], but these data would also serve to further our understanding of the input and distribution of land-based pollution to adjacent coral reefs.

Presently, the wealth of causative knowledge linking localized stressors with reef assemblages has been derived through examining either herbivory *or* water quality [23]–[25], [35], often within no-take marine preserves, or conducted using cage-based, manipulative experiments [36]. For instance, multi-year investigations found that increased herbivory facilitated recovery within a Caribbean no-take marine preserve, evidenced through improved growth rates of corals in the preserves [25], [37]. Similarly, the distribution and density of grazing urchin recovery in the Caribbean was linked with coral recruitment and growth patterns [38], [39]. Smith et al. [23] report that demographic patterns following a bleaching event were most dynamic where poor water quality existed along the Great Barrier Reef. While the present results agree with these findings, they also highlight clear synergistic dependencies that are novel and warrant further attention. To what extent might enhanced herbivory be able to account for reduced water quality, or vice-versa, in order to maintain desirable ecological states?

Synergistic experimental designs (i.e., *water quality × herbivory*) have mainly been constrained to smaller temporal and spatial scales due to logistical considerations, thus establishing a reliance upon manipulative environments [10]. A meta-analysis of cage-based experiments extrapolated that herbivory, acting alone, is typically the greatest predictor of tropical macroalgal dynamics [36]. However, recall that herbivore exclusion plots with continuous nutrient supplementation represent extreme situations, and patterns observed on individual plots of reef may not hold for reefscapes [17]. Here we highlight contrasting findings based upon observations at larger spatial scales, and call for long-term monitoring programs to consider the collection of complimentary environmental datasets to augment our collective insight. Through an improved elucidation of ecological-environmental coupling on coral reefs, management programs will have a better foundation to define and meet their goals.

Coral reef disturbance and recovery cycles are ubiquitous at time scales relevant to the resource needs of Pacific island societies and economies [40], [41]. Efforts to improve preservation and sustainability should build upon describing the relatively well-known patterns behind recovery [42]–[46], and focus more upon predicting why patterns become emergent.

## Materials and Methods

### Ethics Statement

All research was approved by and conducted in collaboration with the American Samoa Environmental Protection Agency.

### Study Location

Data were collected in conjunction with a long-term monitoring program on Tutuila, American Samoa [32], a high volcanic island in the South Pacific (Figure 1). Seventeen monitoring sites have been examined on a rotational basis between 2003 and 2008 based upon logistical constraints and weather patterns (Table S1). Monitoring stations were established on the nearshore reef slopes (8–10 m) adjacent to selected watersheds, approximately 250 m away from stream discharge, collectively representing gradients of environmental regimes. Inherent differences in reef assemblages and geological reef structures exist due to varying physical environments on Tutuila [32], [33]. On Tutuila, two visually distinct reef types categorized by previous studies are relevant: 1) primary framework with interstitial spaces common throughout the reef matrix, found mainly on the south side of Tutuila, 2) primary framework with a well-cemented, underlying basement, lacking significant interstitial spaces, mainly found on the northern side of the island, and 3) intermixed sand and primary-framework reef patches, uncommonly encountered on both sides of the island. Primary coral framework (Holocene) is defined as a consolidated reef matrix created mainly by large coral skeletons cemented together with coralline algae, and interstitial spaces refer to the presence of cavities within the primary reef framework (summarized by [46]). Monitoring designs selected representative sites within each geomorphological class, along gradients of watershed sizes, land-use, and human influences (Table S1). This provides for the partitioning of ecological variance and isolation upon variables of interest, namely pollution proxies and fish abundances.

### Ecological Data

Data collection efforts were designed to enable the detection of trends in resources deemed ecologically and economically valuable by the people (i.e., coral, macroinvertebrates, algae, and fish assemblages) that shift at time scales appropriate to recommend and assess management actions (1–5 years). Benthic cover was evaluated using a modified video belt transect method [47]. For each site, video data were collected along three 50 m transects using an underwater digital video camera that recorded 0.5 m ×50 m belts. These videos were analyzed by extracting 60 individual frames per transect, projecting five randomly situated dots, and noting the life form under each. The benthic categories chosen for analysis were corals (to genus level), turf algae (less than 2 cm), macroalgae (greater than 2 cm, to genus level if abundant), coralline algae known to overgrow coral (i.e., *Peyssonnelia, Pneophyllum*) [48]–[50], other coralline algae, sand, and other invertebrates (genus level if abundant). Besides categorical estimates, a benthic substrate ratio [32], [33] was calculated as the percent cover of coral, soft coral, and coralline algae divided by the percent cover of macroalgae, turf algae, and inhibitive coralline algae.

Coral communities were examined using a point-quadrat technique. Eight, 1×1 m quadrats were tossed at equal distances along each transect lines. Every colony whose center point lay inside the quadrat was recorded to species level, and the maximum diameter and diameter perpendicular to the maximum were measured. These measurements were used to estimate percent coverage, relative abundance, population density, and geometric diameter, with the mathematical assumption that colonies are circular. Species richness per unit area was calculated as the average number of coral species that were found within each quadrat. Margalef's d-statistic was calculated as a measure of the number of corals present, making some allowance for the abundance of individuals, or community evenness [51].

Fish numerical abundance and biomass were estimated using a modified, Bohnsack stationary point count (SPC), with a radius of 7.5 m [52]. At each site, five SPC replicates were conducted. All large fish (>20 cm TL) as well as all fish known to be exploited for either commercial or artisanal fisheries were surveyed. All fish were identified to species level, counted, and size estimates to the nearest 5 cm were recorded for each individual observed within the SPC boundary during a 5 minute observation period. Fish biomass estimates were calculated from the lengths recorded using the formula W = A*L∧B where W = weight, L =  length, and A&B =  growth parameters obtained from fishbase [53]. Fish were assigned trophic groups based upon published accounts of diet studies. For the purposes of this study, only herbivorous fish species were considered; inclusive of scrapers, excavators, and detritivores [54]. In total, 44 species of herbivorous fishes consisting primarily of surgeonfishes (Acanthuridae) and parrotfishes (Scaridae), along with a few species of angelfishes (Pomacanthidae), rabbitfishes (Siganidae), and rudderfishes (Kyphosidae), were considered for analyses.

Macroinvertebrates were counted along three 50×4 m transects at each site, identified to the genus level.

### Environmental Data

Water quality data originated from the ASEPA watershed monitoring program, and have been collected from most major watersheds on a rotational basis since 2004. For the purposes of this study a water quality index was created for each watershed based upon bacteria and nutrient concentrations (NO_2_ + NO_3_, NH_4_, PO_4_, Total P, and Total N). The index represents mean, ranked values for these constituents.

Wave-exposure data were gathered from NOAA Wave Watch III model predictions, summarized for American Samoa [31]. For each monitoring site, mean wave heights were recorded with respect to their angle of exposure, using the wave-rose data. Shortly after data collection efforts began, a category 5 cyclone (Heta) impacted much of the region, and wave heights reaching as high as 13 m were reported offshore. The suspected drivers of the ubiquitous decline in coral cover surrounding this time frame are the direct impacts of the cyclone, major upwelling of cool nutrient rich waters that accompanies tropical storms [55], or time-integrated responses of both [56].

Watershed statistics were derived from existing American Samoa Department of Commerce GIS layers [57], while land-use data were derived from the United States Forest Service vegetation maps [58]. “Disturbed land” represented all land use categories that were not classified as tropical forests within each watershed, including urban development, agriculture, savannah, shrub, and grassland.

Human population estimates were derived from the most recent census report, while pig population data were collected during American Samoa Environmental Protection Agency household inspections from June to October, 2006. These data were used as proxies of water quality for multiple regression analyses encompassing the entire island, as water quality data were not ubiquitously available.

### Data Analysis

First, a widespread coral mortality event during the austral summer of 2003, coincident with Cyclone Heta, was characterized. Comparisons before and after the event were made for benthic and coral assemblage data using standard pairwise testing procedures, assumptions, and transformations when appropriate. Subsequently, coral assemblages were examined in multivariate space using principle components analyses (PCA's) [59] to provide further insight into site-specific changes, and different ecological recovery patterns (2003–2007). Species-centered PCA's rotate the multidimensional species similarity matrices to extract as much variance as possible (i.e., show the greatest gradients) in two dimensions. The resultant eigenvalues describe how much of the ecological variance was attributed to each axis.

Second, four sites where data were available before and after the disturbance event, and that held similar initial coral reef assemblages, were grouped based upon their differential dynamics through time as: 1) ‘high-recovery reefs’, where mean coral species richness and benthic substrate ratios remained statistically similar, and 2) ‘low-recovery reefs’, where both metrics had significant declines. The ecological metrics used in these classifications were selected based upon their documented sensitivity to localized stressors [60], while being less influenced by natural disturbances [4]. Coral species richness patterns have previously been predicted by gradients of human influence [4], [33], whereby pollution and/or reduced herbivory facilitated selective environmental conditions that were corroborated with a narrowing of the local species pool as disturbance-recovery cycles become evident. Favorable benthic substrates, defined above, have been corroborated by numerous studies that show relationships between the noted benthic categories, high coral recruitment, and benign interactions with adult colonies [40], [48]–[50]. Clearly reef assemblages from different locales differ to some extent, and evaluating ‘status’ without knowing individual disturbance histories presents a challenge. Here, we address this by focusing upon rates of change in standardized ‘status’ metrics rather than absolute values.

Water quality, herbivory, and an interaction term were compared with respect to recovery status using similar pairwise testing procedures and assumptions noted above. Marine water quality datasets originated from collection events between 2004 and 2007. Herbivorous fish data were from 2007 and 2008. Prior to examination, both were standardized across all sites residing in similar geological settings to provide for equal weighting. The interaction term was calculated by randomly pairing each replicate herbivorous fish estimate with a measure of water quality. This process was iterated for all possible combinations of herbivory and water quality data, to ensure that the distribution of the interactive term variable was normal, and the appropriate mean and variance estimates were used.

Third, multiple regression models were created to test for consistencies between site-specific findings regarding ecological recovery and island-wide patterns. These tests examined interdependencies between ecological metrics (coral cover, species richness, and benthic substrate ratio) and localized stressors along a gradient of 17 sites. Data handling included: 1) creating site-based averages for all years, 2) stratifying data by reef type, 3) standardizing independent data, 4) conducting power-transformations on the data if normality assumptions were not met [61], and 5) constructing models that defined what combination of environmental variables best predicted the ecological variance (R-statistical package, [62]). Independent variables consisted of watershed area, “disturbed land”, wave exposure, human population, pig population, and herbivorous fish density. This study examined the best fit models using Akaike's Information Criterion; briefly, the selected models explained the greatest proportion of the variance while using the least number of explanatory variables to ensure the greatest precision, accuracy, and repeatability.

Based upon significant trends found, interpolation plots were created to visualize island-wide patterns between gradients of localized stressors and resiliency status. Recovery status was defined categorically above, however, here the continuous dependent variable was calculated by combining standardized scores of coral species richness and benthic substrate ratio's.

## Supporting Information

Table S1 Environmental characteristics associated with each site, numbers in parentheses refer to site location (Fig. 1). Reef types are categorized as follows: 1) primary framework reefs with interstitial porosity, common to the south side of Tutuila, 2) primary framework reefs with a well-cemented basement, common to the north side, and 3) sand and patches of reef.
(0.12 MB DOC)

Figure S1 Results from pairwise testing of benthic substrate ratios for four sites where coral assemblages were initially similar but varied in the years following cyclone Heta. Local site names and site numbers referring to Figure 1 are shown. P-values are as follows: (*P<0.05, **P<0.01, and ***P<0.001).
(0.84 MB EPS)

Figure S2 Coral colony size-class distributions for four sites where coral assemblages were initially similar but varied in the years following cyclone Heta. At site 10 there was a significant change in colony size (P<0.05, Kolmogorov-Smirnov cumulative frequency test), while at site 11 there was a non-significant shift in colony size to larger, mainly Porites, corals (see Fig. 2b).
(1.50 MB EPS)
