# Supplementary material for: Water Quality and Herbivory Interactively Drive Coral-Reef Recovery Patterns in American Samoa
Source: PLoS One. 2010 Nov 10;5(11):e13913. doi: 10.1371/journal.pone.0013913 (PMC2978088; doi:10.1371/journal.pone.0013913)
Supplement: Table S1 — Environmental characteristics associated with each site, numbers in parentheses refer to site location (Fig. 1). Reef types are categorized as follows: 1) primary framework reefs with interstitial porosity, common to the south side of Tutuila, 2) primary framework reefs with a well-cemented basement, common to the north side, and 3) sand and patches of reef. (0.12 MB DOC) [file pone.0013913.s001.doc]

| Site | Years Surveyed | Reef Type | Watershed Size (km^2^) | Disturbed Land (km^2^) | Human Population | Pig Population | Wave Height (m) |
| --- | --- | --- | --- | --- | --- | --- | --- |
| Alega (8) | 03’, 05’, 08’ | 1 | 0.96 | 0.03 | 111 | 14 | 0.70 |
| Alofau (10) | 03’, 07’ | 2 | 1.08 | 0.13 | 495 | 97 | 0.67 |
| Aoa (11) | 03’, 07’ | 2 | 2.15 | 0.08 | 507 | 106 | 0.46 |
| Fagaalu (6) | 03’, 05’, 08’ | 1 | 2.46 | 0.29 | 1006 | 65 | 0.70 |
| Fagafue (17) | 07’ | 2 | 3.56 | 0.3 | 1142 | 67 | 0.40 |
| Fagaitua (9) | 03’, 07’ | 1 | 1.46 | 0.09 | 483 | 76 | 0.67 |
| Fagasa (16) | 05’, 08’ | 2 | 3.47 | 0.21 | 900 | 104 | 0.46 |
| Fagatele (4) | 05’, 08’ | 1 | 0.49 | 0.06 | 0 | 0 | 0.61 |
| Laulii (7) | 05’, 08’ | 1 | 1.76 | 0.23 | 1186 | 158 | 0.70 |
| Leone (2) | 03’, 05’, 08’ | 1 | 4.75 | 0.92 | 2200 | 429 | 0.67 |
| Masausi (12) | 05’ | 2 | 0.8 | 0.04 | 192 | 52 | 0.40 |
| Masefau (13) | 03’, 07’ | 2 | 3.21 | 0.11 | 435 | 89 | 0.37 |
| Matuu (5) | 07’ | 1 | 1.2 | 0.13 | 671 | 44 | 0.70 |
| Nua Seetaga (1) | 08’ | 3 | 1.76 | 0.42 | 313 | 238 | 0.67 |
| Tafeu (15) | 05’, 08’ | 2 | 0.92 | 0.001 | 0 | 0 | 0.46 |
| Vatia (14) | 07’ | 3 | 3.61 | 0.16 | 648 | 43 | 0.34 |
| Vaitogi (3) | 07’ | 1 | 1.61 | 0.25 | 0 | 0 | 0.70 |
